# The Calcium Sensor Calcineurin B-Like Proteins -Calcineurin B-Like Interacting Protein Kinases Is Involved in Leaf Development and Stress Responses Related to Latex Flow in *Hevea brasiliensis*

**DOI:** 10.3389/fpls.2022.743506

**Published:** 2022-02-25

**Authors:** Xiaohu Xiao, Chunyan Mo, Jinlei Sui, Xianzu Lin, Xiangyu Long, Yunxia Qin, Yongjun Fang, Chaorong Tang

**Affiliations:** ^1^Rubber Research Institute, Chinese Academy of Tropical Agricultural Sciences, Haikou, China; ^2^College of Tropical Crops, Hainan University, Haikou, China; ^3^Natural Rubber Cooperative Innovation Center of Hainan Province and Ministry of Education of PRC, Haikou, China; ^4^Public Research Laboratory, Hainan Medical University, Haikou, China

**Keywords:** *Hevea brasiliensis*, calcineurin B-like protein, CBL-interacting protein kinase, gene expression, protein interaction, stress, latex flow

## Abstract

Latex flow in *Hevea brasiliensis* (the Para rubber tree), the sole commercial source of natural rubber (*cis*-1,4-polyisoprene, NR), renders it uniquely suited for the study of plant stress responses. Calcineurin B-like interacting protein kinases (CIPK) serving as calcium-sensor protein kinases react with calcineurin B-like proteins (CBL) to play crucial roles in hormone signaling transduction and response to abiotic stress in plant developmental processes. However, little is known about their functions in *Hevea*. In this study, a total of twelve *CBL* (*HbCBL*) and thirty *CIPK* (*HbCIPK*) genes were identified from the *Hevea* genome. Structure and phylogenetic analysis assigned these CIPKs to five groups and CBLs to four groups, and mapped onto fourteen of the eighteen *Hevea* chromosomes. RNA-seq and qPCR analysis showed that the expressions of *HbCBL* and *HbCIPK* genes varied in the seven *Hevea* tissues examined, i.e., latex (cytoplasm of rubber-producing laticifers), bark, leaf, root, seed, female flower, and male flower. The expressions of two *HbCBL* and sixteen *HbCIPK* genes showed upward trends during leaf development. Following ethylene yield stimulation and the latex tapping treatment, both practices invoking stress, the expression levels of most latex-expressed genes were significantly altered. Yeast two-hybrid test revealed interactions for multiple combinations of HbCBLs and HbCIPKs with substantial gene expression in latex or other *Hevea* tissues. However, all the HbCBL-HbCIPK complexes examined did not recruit HbSOS1 or AtSOS1 to form functional salt tolerance SOS pathway in yeast cells. Taken together, the results suggested a role of the *Hevea* CBL-CIPK network as a point of convergence for several different signaling pathways in growth, development, and stress responses in relation to latex production.

## Introduction

Calcium, functioning as a messenger of plant cells, mediates a multitude of plant responses to external stimuli and regulates a wide range of physiological processes (Gilroy and Trewavas, 2001). Calcium-binding proteins, such as calcineurin B-like (CBL) proteins, represent important relays in plant calcium signaling. These proteins form a complex network with their target kinases, i.e., the CBL-interacting protein kinases (CIPKs) (Gong et al., 2004; Boudsocq and Laurière, 2005; Weinl et al., 2009). The CIPK protein consists of an N-terminal protein kinase domain and a C-terminal autoinhibitory domain known as the NAF or FISL motif, with CBLs interacting with CIPKs through the C-terminal domain that is conserved among different CIPKs (Shi et al., 1999; Guo et al., 2001; Luan, 2009).

Calcineurin B-like interacting protein kinases are extensively involved in plant stress responses. Physiological roles of CBL and CIPK were firstly uncovered in the salt overly sensitive (SOS) pathway, with SOS3 (CBL4) and SOS2 (CIPK24) shown to synergistically up-regulate the activity of the Na^+^/H^+^ exchanger SOS1 in Arabidopsis roots, thus leading to Na^+^ efflux from cells in high-salt environment and an enhanced salt detoxification process (Shi et al., 2000; Viswanathan et al., 2004). In Arabidopsis, CBL10 (SCaBP8) that interacts with CIPK24/SOS2 is expressed almost exclusively in the shoots and leaves, and functions in the transport of salt into vacuoles, and control of cellular salt homeostasis (Kim et al., 2007; Quan et al., 2007). CBL1 and CBL9, both interacting with CIPK23, regulate potassium (K) uptake and stomatal movements in leaf transpiration and root potassium uptake in Arabidopsis (Li et al., 2006; Xu et al., 2006; Lee et al., 2007; Cheong et al., 2010). CBL2 and CBL7 both interact with CIPK11, but play different regulatory roles in plasma membrane H^+^-ATPase activity (Fuglsang et al., 2007; Yang et al., 2019). Besides Arabidopsis, the studies on CBLs and CIPKs have been widely reported in other plant species, such as *Oryza sativa* (Kolukisaoglu et al., 2004; Xiang et al., 2007), *Populus trichocarpa* (Zhang et al., 2008), *Manihot esculenta* (Hu et al., 2015; Mo et al., 2018), *Brassica napus L.* (Zhang et al., 2014), *Pyrus bretschneideri* (Tang J. et al., 2016), *Physcomitrella patens* (Kleist et al., 2014), pointing to their important and conserved roles in the regulation of abiotic stresses, hormone signaling and intrinsic developmental programming in plant growth and development. Nevertheless, the identities and functions of the CBL and CIPK family genes in *Hevea brasiliensis* are still unknown.

Natural rubber in *Hevea brasiliensis* is synthesized and stored in the laticifer cells which are differentiated from the cambium and arranged in concentric rings (when viewed in cross section) in the phloem region (Tupy, 1989; Hao and Wu, 2000). The bark of the rubber tree is excised every 2 to 3 days to sever the laticifer rings in a process called tapping to enable the outflow of latex (Tupy, 1989). At each tapping, several tens to a few hundred milliliters of latex per tree are expelled from the laticifers and harvested for sustainable rubber production. Application of ethylene gas or ethephon (2-chloroethylphosphonic acid, an ethylene generator) to the trunk bark of the rubber tree can significantly increase rubber yield. However, the underlying mechanisms in ethylene stimulation are not yet fully understood, although ethylene signaling and response are assumed to play critical roles (Tang C. et al., 2016). The laticifer network could serve as a useful system for the study of stress and signal transmission.

In this study, the *Hevea* CBL and CIPK genes were identified by genome-wide analysis, and their gene structure, phylogeny and chromosomal distribution were analyzed. Based on RNA-seq data and qRT-PCR analysis, the *HbCIPK* and *HbCBL* gene expression profiles in different *Hevea* tissues and leaf development stages were determined. The influence of ethylene stimulation and the onset of tapping on their expression levels were also analyzed. Furthermore, the interaction relationships between HbCBL and HbCIPK proteins were detected by yeast two-hybrid, and the salt tolerance SOS pathway in *Hevea* was investigated by yeast complementation test. Our aim was to understand the roles of the CBL-CIPK network in *Hevea* responses to abiotic stress, ethylene-based latex flow stimulation and leaf development.

## Results

### Genome-Wide Identification of Calcineurin B-Like Interacting Protein Kinases and Calcineurin B-Like Proteins Family Genes in *Hevea brasiliensis*

BLAST and Hidden Markov Model searches were conducted to identify *H. brasiliensis* CIPKs and CBLs using Arabidopsis, rice and poplar CIPK and CBL protein sequences as queries. A total of twelve CBLs (named *HbCBL1* to *12*) and thirty CIPKs (named *HbCIPK1* to *30*) were identified from the *H. brasiliensis* genome (Tang C. et al., 2016). Detailed information, including protein length, isoelectric point (pI), molecular weight (MW), number of introns and evolution group of identified HbCBLs and HbCIPKs, is listed in Table 1. The number of amino acid residues of the identified HbCIPKs ranged from 328 (HbCIPK22) to 541 (HbCIPK17), and HbCBLs ranged from 165 (HbCBL2) to 247 (HbCBL5). Their relative molecular mass ranged from 37.29 kDa (HbCIPK22) to 61.30 kDa (HbCIPK17) for HbCIPKs and 19.38 kDa (HbCBL2) to 28.44 kDa (HbCBL5) for HbCBLs. The isoelectric points of HbCBLs and HbCIPKs were between 4.64 (HbCBL6) and 9.21 (HbCBL2), and between 5.47 (HbCIPK28) and 9.51 (HbCIPK22), respectively. It is noteworthy here that the isoelectric point of HbCBL2, at 9.21, was an outlier that was significantly higher than those of the other HbCBLs (Table 1). Conserved domain analysis confirmed that most of the HbCIPKs identified harbored the Pkinase (PF00069) and NAF (PF03822) domains, while all the HbCBLs harbored the calcium-binding EF hand domains (PF13833 and PF13499) which are the hallmark of CBL family.

**TABLE 1:** Characteristics of *CIPK* and *CBL* genes in *Hevea brasiliensis.*

Gene*s*	ID	CDS length in bp	Predicted protein			No. of introns	Group
			Length (aa)	isoelectric point	Mol Wt		
*HbCIPK1*	scaffold0014_83038	1,317	439	6.35	49874.40	13	A
*HbCIPK2*	scaffold0016_914468	1,311	437	9.22	49190.93	0	C
*HbCIPK3*	scaffold0050_2486504	1,176	392	6.01	44145.38	0	E
*HbCIPK4*	scaffold0050_2491974	1,302	434	8.47	49318.37	1	C
*HbCIPK5*	scaffold0050_2493973	1,359	453	9.03	51228.77	0	C
*HbCIPK6*	scaffold0069_353896	1,338	446	6.93	50657.28	13	A
*HbCIPK7*	scaffold0099_255532	1,338	446	7.62	50550.11	13	A
*HbCIPK8*	scaffold0140_117108	1,425	475	7.89	53522.25	14	A
*HbCIPK9*	scaffold0163_468878	1,293	431	8.78	48708.09	12	A
*HbCIPK10*	scaffold0181_102123	1,431	477	6.49	54321.69	13	A
*HbCIPK11*	scaffold0198_1059208	1,335	445	8.82	50240.9	14	A
*HbCIPK12*	scaffold0387_428199	1,506	502	8.8	56294.84	0	C
*HbCIPK13*	scaffold0548_269862	1,290	430	9.33	48130.81	0	B
*HbCIPK14*	scaffold0696_308862	1,296	432	8.59	49097.59	0	D
*HbCIPK15*	scaffold0696_410579	1,377	459	8.72	52133.09	0	C
*HbCIPK16*	scaffold0703_504618	1,293	431	6.81	48162.36	11	A
*HbCIPK17*	scaffold0724_249981	1,623	541	8.81	61301.19	16	A
*HbCIPK18*	scaffold0844_55952	1,371	457	8.83	51759.92	0	C
*HbCIPK19*	scaffold0888_27409	1,287	429	8.9	48325.6	0	D
*HbCIPK20*	scaffold0942_13608	1,452	484	7.15	54429.52	0	E
*HbCIPK21*	scaffold1198_176987	1,314	438	9.11	49051.66	0	B
*HbCIPK22*	scaffold1299_99244	984	328	9.51	37287.59	0	C
*HbCIPK23*	scaffold1550_32944	1,314	438	9.16	49518.15	0	C
*HbCIPK24*	scaffold1903_22786	1,344	448	8.75	50436.16	0	C
*HbCIPK25*	scaffold1947_8598	1,380	460	7.96	51792.84	1	C
*HbCIPK26*	scaffold2416_1776	1,422	474	8.73	53758.12	0	C
*HbCIPK27*	scaffold2591_4435	1,533	511	9.19	57653.84	14	A
*HbCIPK28*	scaffold2753_17813	1,074	358	5.47	40331.28	0	C
*HbCIPK29*	scaffold2753_19142	1,371	457	6.5	51671.65	0	C
*HbCIPK30*	scaffold2989_13981	1,251	417	8.9	47133.5	13	A
*HbCBL1*	scaffold0196_591691	669	223	4.76	25779.37	7	II
*HbCBL2*	scaffold0246_1253718	495	165	9.21	19378.34	2	IV
*HbCBL3*	scaffold0407_1051914	657	219	5.07	25129.94	7	IV
*HbCBL4*	scaffold0407_1073206	576	192	4.66	21977.03	7	IV
*HbCBL5*	scaffold0578_391501	741	247	4.66	28438.53	8	I
*HbCBL6*	scaffold0578_405713	741	247	4.64	28343.49	8	I
*HbCBL7*	scaffold0629_587305	654	218	4.9	25193.01	7	IV
*HbCBL8*	scaffold0706_448477	522	174	5.17	20076	6	I
*HbCBL9*	scaffold0782_201099	648	216	4.98	24903.48	7	IV
*HbCBL10*	scaffold0884_405775	639	213	4.66	24482.87	7	III
*HbCBL11*	scaffold1483_74222	669	223	4.76	25793.38	7	II
*HbCBL12*	scaffold2093_21166	639	213	4.7	24249.6	7	III

### Phylogenetic and Gene Structure Analysis of the HbCIPK and HbCBL Family Genes

**FIGURE 1:**
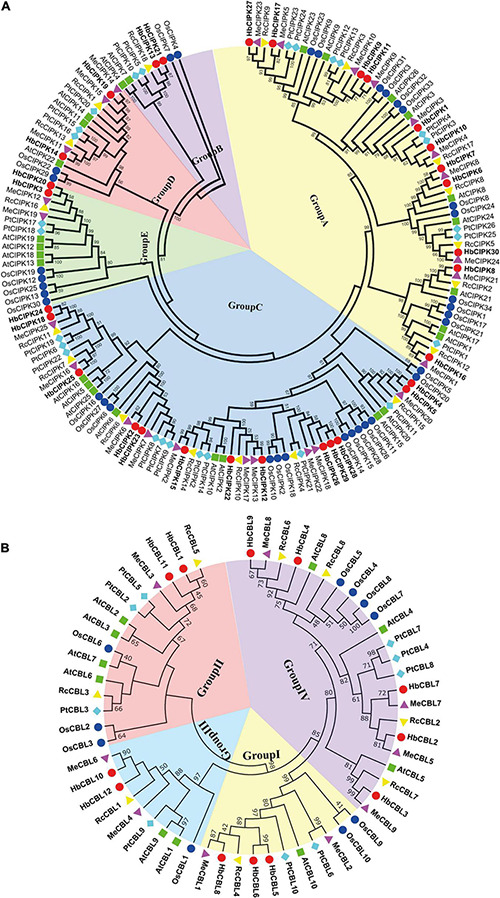
Phylogenetic analysis of *HbCIPK* and *HbCBL* genes in *Hevea brasiliensis* and five other plant species. Unrooted phylogenetic trees of plant HbCIPK and HbCBL proteins were constructed using the neighbor-joining method with the MEGA 6.0 program. **(A)** The plant species and their CIPK proteins are as follows: *H. brasiliensis*, HbCIPKs (30), marked with red dots; *A. thaliana*, AtCIPKs (26); *P. trichocarpa*, PtCIPKs (26); *O. sativa*, OsCIPKs (34); *M. esculenta*, MeCIPKs (25); *R. communis*, RcCIPKs (18). **(B)** The plant species and their CBL proteins are as follows: *H. brasiliensis*, HbCBLs (12), marked with red dots; *A. thaliana*, AtCBLs (10); *P. trichocarpa*, PtCBLs (9); *O. sativa*, OsCBLs (10); *M. esculenta*, MeCBLs (9); *R. communis*, RcCBLs (8).

**FIGURE 2:**
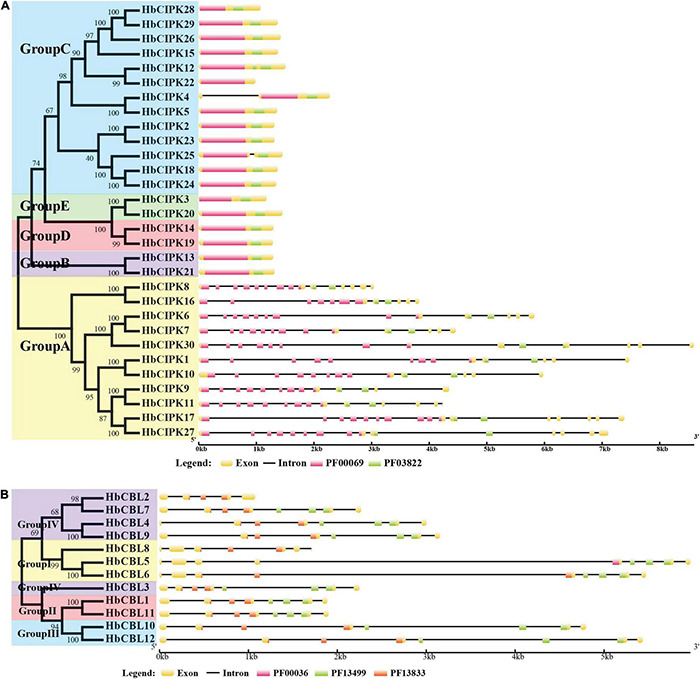
Structural organization of *HbCIPK* and *HbCBL* genes in *Hevea brasiliensis*. **(A)** Structural organization of *HbCIPK* genes in *H. brasiliensis.* **(B)** Structural organization of *HbCBL* genes in *H. brasiliensis.* Exons and introns are represented by boxes and black lines, respectively, with the sizes of the exons and introns proportional to their sequence lengths. The Pkinase domain (PF00069) is represented by pink boxes, the NAF domain (PF03822) is represented by green boxes in 2A, while the EF hand domains (PF00036, PF13499 and PF13833) are represented by pink, green, and red boxes, respectively, in 2B.

Phylogenetic analysis of the thirty HbCIPKs, together with 103 CIPKs from five other plants, classified the CIPK family into five clusters (Group A to E, Figure 1A and Supplementary Tables 1, 2). There were eleven HbCIPK members (HbCIPK1, 6–11, 16–17, 27 and 30) in Group A, thirteen (HbCIPK2, 4–5, 12, 15, 18, 22–26, 28–29) in Group C, and two each in the other three groups, i.e., HbCIPK13 and 21 in Group B, HbCIPK14 and 19 in Group D, and HbCIPK3 and 20 in Group E. As expected, CIPKs from *Hevea* generally had closer relationships with those from the two Euphorbiaceae family species, *Manihot esculenta* and *Ricinus communis*, than those from Arabidopsis, rice or poplar, in accordance with plant taxonomic status. Phylogenetic analysis based on predicted amino acid sequences identified some closely related pairs of HbCIPKs, such as HbCIPK9 and 11 in Group A, HbCIPK13 and 21 in Group B, HbCIPK2 and 23, HbCIPK4 and 5, HbCIPK18 and 24, and HbCIPK26, 28 and 29 in Group C, and HbCIPK3 and 20 in Group E (Figure 1A). Similar trends are also observed in other plant species (Hu et al., 2015), and closely related HbCIPK pairs often have similar cellular localization and functions. The exon-intron structures of the thirty *HbCIPK* genes were determined based on their predicted genomic sequences. As shown in Figure 2A, most *HbCIPK* members within the same groups shared similar gene structure in terms of intron number, domain localization and exon length. It is worth noting that the members in Group A had eleven to sixteen introns, while those in the four other groups had no intron or only one. A similar intron-rich or poor pattern is also observed in the *CIPKs* from other plant species, such as Arabidopsis, rice, poplar, cassava, pear, and soybean (Kolukisaoglu et al., 2004; Zhang et al., 2008; Hu et al., 2015; Tang J. et al., 2016; Zhu et al., 2016), reflecting a conserved feature of the *CIPK* family in gene structure. Considering the rate of intron loss is faster than that of intron gain after segmental duplication (Roy and Penny, 2007), the *CIPKs* of group A might represent the progenitor genes of the *CIPK* family.

**FIGURE 3:**
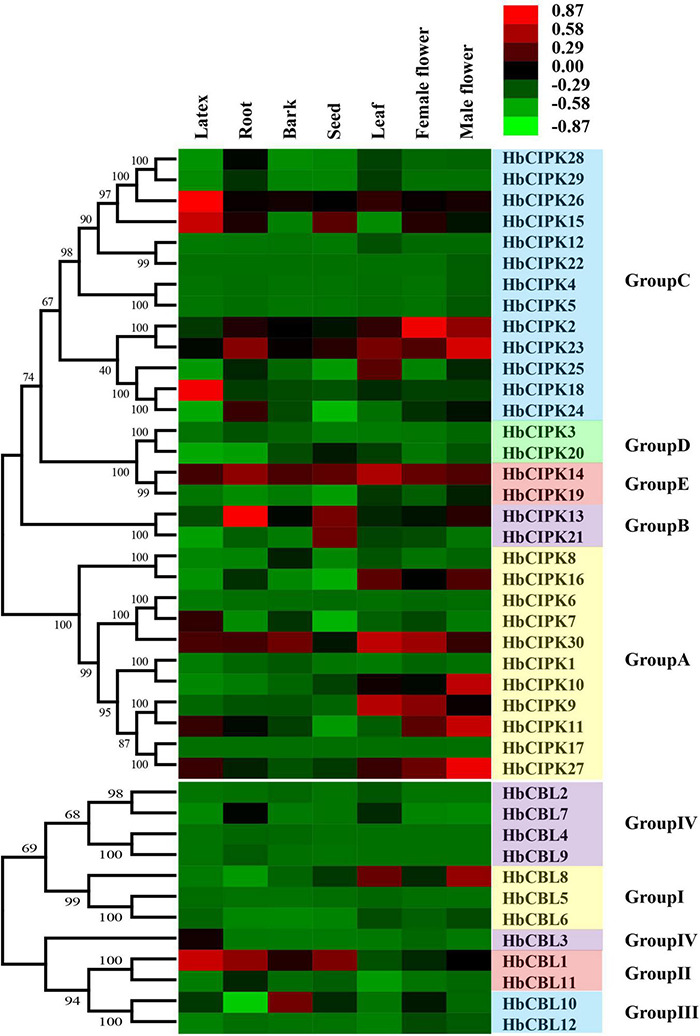
Expression analyses of *HbCIPK* and *HbCBL* genes in different tissues based on Solexa sequencing. Phylogenetic clustering and differential expression analysis of *HbCIPK* and *HbCBL* genes in seven tissues (leaf, bark, latex, root, seed, female flower, and male flower, Project Accession: PRJNA310171).

Phylogenetic analysis put the *CBL* genes of *Hevea* and the other five plants into four groups (Group I to IV, Figure 1B), with three, two, two and five members in Group I, II, III, and IV, respectively. This analysis also identified six closely related orthologous pairs between *Hevea* and cassava (HbCBL9 and MeCBL8, HbCBL7 and MeCBL7, HbCBL2 and MeCBL5, HbCBL3 and MeCBL9, HbCBL8 and MeCBL1, HbCBL10 and MeCBL6), suggesting the existence of an ancestral set of *CBL* genes prior to the divergence of *Hevea* and cassava. The results of gene expression analysis (Figure 3) showed that HbCBL1, 8 and 10 might be the main direction of HbCBLs evolution. Our analysis of genomic organization of the *HbCBLs* revealed that eight of the twelve members contained seven introns, two contained eight introns, one contained four introns and one contained six introns (Figure 2B). Except for *HbCBL2* and *HbCBL8*, most *HbCBL* members within the same groups shared very similar gene structures in terms of intron number, domain localization, and exon length.

### Chromosomal Distribution and Colinearity Analysis of the HbCIPK and HbCBL Family Genes

**FIGURE 4:**
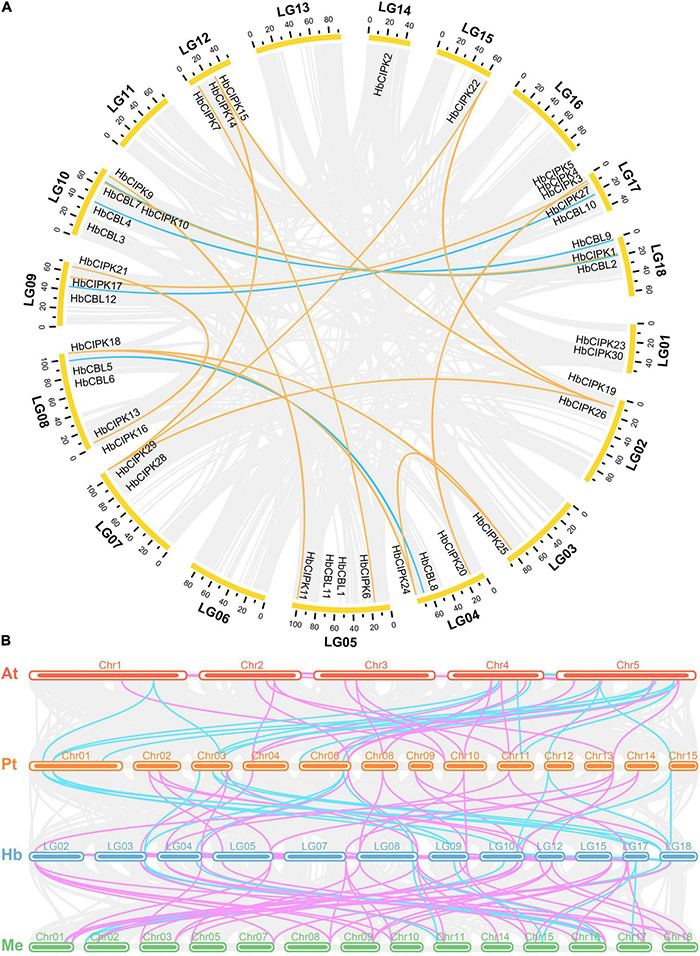
Chromosomal location and colinearity of *CIPK* and *HbCBL* genes in *Hevea* and three other plant genomes. **(A)** All the Hevea CBL and CIPK family genes are depicted in the *Hevea* chromosomes (LG1-18). Gray background lines indicate collinear blocks in whole *Hevea* genome, and the collinear relationships of *HbCBL* and *HbCIPK* genes are indicated by solid color lines. **(B)** Colinearity analysis of CBL and CIPK genes in four plant species, i.e., At (*A. thaliana*), Pt (*P. trichocarpa*), Hb (*H. brasiliensis*) and Me (*M. esculenta*). Gray background lines indicate collinear blocks between different plant genomes. Colinear CBL and CIPK genes are indicated by solid color lines (light blue, CBL; pink, CIPK).

The locations of *HbCIPK* and *HbCBL* genes in *Hevea* chromosomes were as shown in Figure 4A. The *HbCIPK* genes were mapped onto fourteen of the eighteen *Hevea* chromosomes, while the *HbCBL* genes were located on seven chromosomes. However, distribution of these two sets of genes on the chromosomes was uneven. There was no *HbCIPK* or *HbCBL* members mapped onto chromosomes 6, 11, 13, and 16, whereas the other chromosomes contained one to five members. Chromosome location analysis also found some tandem repeat genes, such as *HbCIPK19* and *26, HbCIPK28* and *29, HbCIPK14* and *15, HbCIPK3, 4* and *5*, which were located on chromosomes 2, 7, 12, and 17, respectively. The same was true of *HbCBL1* and *11, HbCBL5* and *6, HbCBL3* and *4* located on chromosome 5, 8, and 10, respectively. Collinearity analysis found some *HbCIPK* members might have been amplified by chromosome duplication, such as in the case of *HbCIPK26* and *28, HbCIPK24* and *18, HbCIPK11* and *9, HbCIPK13* and *21, HbCIPK6* and *7, HbCIPK20* and *3, HbCBL5 and 8, HbCBL10 and 12, HbCBL4 and 9, HbCBL2 and 7.* This was consistent with their clustering patterns shown in the phylogenetic tree (Figures 1A, 2). Chromosomal location and colinearity analysis of CBL and CIPK genes within *Hevea* (Figure 4A) and across *Hevea* and three other plant species (Figure 4B) were investigated in order to explore the potential evolutionary relationships. The results revealed a higher homology between *H. brasiliensis*, *M. esculenta* and *P. trichocarpa* than that between *P. trichocarpa* and *A. thaliana*. Some HbCIPK and HbCBL genes were collinear with CIPK and CBL genes in *P. trichocarpa*, *A. thaliana* and *M. esculenta*, suggesting their important roles in plant evolution. These results can be useful for subsequent comparative studies of CIPK and CBL genes with known functions.

### Expression Analysis of HbCIPK and HbCBL Genes in Different Tissues

To study the expression profiles and deduce the gene function of HbCBL-HbCIPKs, transcriptome analyses were conducted in seven *Hevea* tissues: latex, bark, leaf, root, seed, and female and male flowers using Solexa sequencing and quantitative PCR as described previously (Xiao et al., 2017). As shown in Figure 3, transcripts of thirteen *HbCIPK* genes (*HbCIPK1, 3–6, 8, 12, 17, 19–20, 22*, and *28*–*29*) and six *HbCBL* genes (*HbCBL2, 4–6* and *12*) were barely detectable in almost all the tissues examined, indicating the loss of their functions during evolution. In contrast, *HbCIPK14, 23, 30* and *HbCBL1* were expressed in most of the tissues examined, indicating their functional conservation in the course of evolution. Some of the *HbCIPK* and *HbCBL* genes displayed preference expression in specific tissues, such as *HbCIPK15, 18* and *26* and *HbCBL1* and *3* in latex, *HbCIPK13*-*14* and *23* and *HbCBL1* in the root, *HbCIPK14* and *30* and *HbCBL10* in the bark, *HbCIPK9, 14* and *30* and *HbCBL8* in the leaf, and *HbCIPK13-15* and *21* and *HbCBL1* in the seed. It is interesting to note that some of the *HbCIPK* and *HbCBL* members showed distinct expression patterns in female and male flowers. For example, the expression levels of *HbCIPK2, 9*, and *30* and *HbCBL10* were much higher in the female flowers than in the male flowers. On the other hand, *HbCIPK10-11, 16, 23* and *27* and *HbCBL1* and *8* were much higher expressed in the male flowers than the female flowers. The expressions of randomly selected *HbCIPK and HbCBL* genes including *HbCBL3* and *8* and *HbCIPK14, 16, 23* and *27* were further investigated by qPCR (Supplementary Figure 1), revealing patterns basically consistent with transcriptome analysis.

### Expression Analysis of HbCIPK and HbCBL Genes in Leaf Development

**FIGURE 5:**
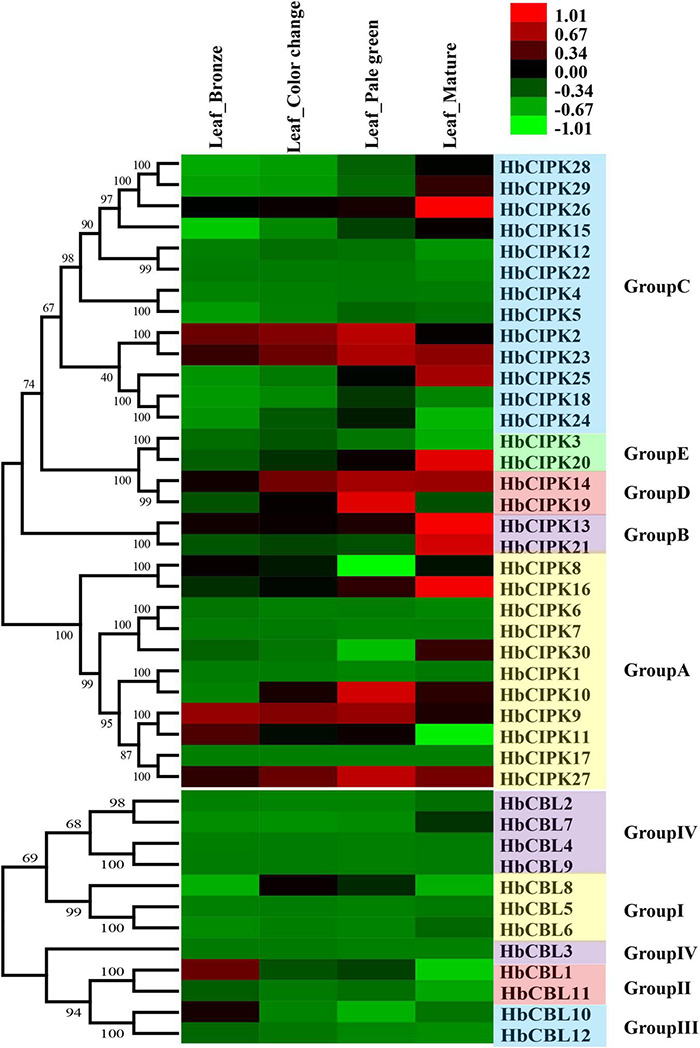
Expression analyses of *HbCIPK* and *HbCBL* genes at different developmental stages of the leaf based on Solexa sequencing. Phylogenetic clustering and differential expression analysis of *HbCIPK* and *HbCBL* genes at four developmental stages of leaves (bronze, color change, pale-green and mature, Project Accession: PRJNA310171).

To obtain information on the functions of *HbCIPK* and *HbCBL* genes in the course of leaf development, we examined their expression levels by RNA-seq at four progressive leaf stages (bronze, color change, pale-green, and mature). As shown in Figure 5, some of the *HbCIPK* genes, i.e., *HbCIPK13-14, 16, 20–21, 25–26*, and *30*, were obviously up-regulated during leaf development. Of the *HbCBL* genes, only *HbCBL7* was similarly up-regulated with leaf development. Some *HbCIPK* and *HbCBL* genes were up-regulated during the first three leaf stages (bronze, color change, and pale-green) but down-regulated toward leaf maturity, as seen for *HbCIPK2, 10, 19, 23*, *27* and *HbCBL8*. A small number of *HbCIPK* and *HbCBL* genes were down-regulated during leaf development, such as *HbCIPK11* and *HbCBL1* and *10.* Some closely related gene pairs exhibited similar expression profiles. For example, *HbCIPK2* and *23*, *HbCIPK28* and *29*, *HbCIPK13* and *21*, *HbCIPK9* and *11* had similar expression patterns during leaf development from soft young leaves to fully hardened mature leaves. Nevertheless, there are more pairs of closely related genes showed dissimilar expression patterns. Examples of these were *HbCIPK1* and *10*, *HbCIPK3* and *20, HbCIPK8* and *16, HbCIPK14* and *19, HbCIPK17* and *27, HbCBL1* and *11, HbCBL2* and *7, HbCBL10* and *12.* The expressions of *HbCIPK14-16, 23, 26* and *27* were further investigated by qPCR (Supplementary Figure 2), all of which were obviously up-regulated during leaf development, consistent with the results of transcriptome analysis.

### Expression Analysis of HbCIPK and HbCBL Genes Following Ethephon Treatment

**FIGURE 6:**
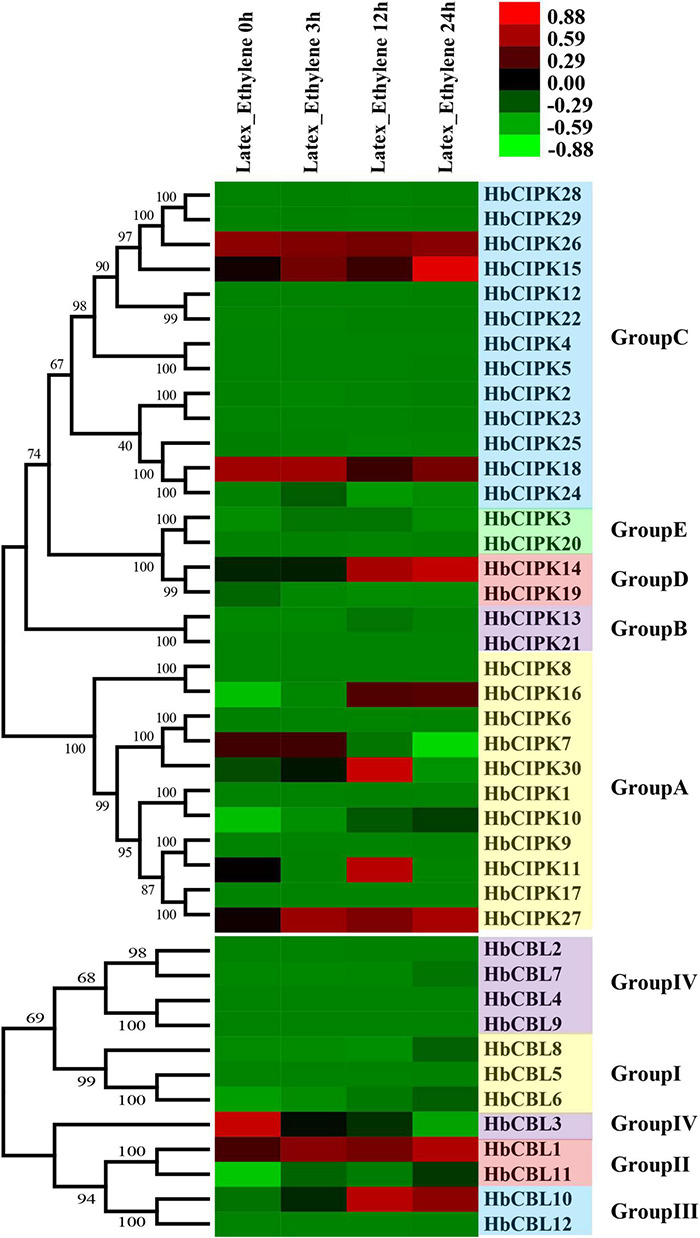
Expression analyses of *HbCIPK* and *HbCBL* genes following ethephon treatment, based on Solexa sequencing. Phylogenetic clustering and differential expression analysis of *HbCIPK* and *HbCBL* genes at different durations following ethephon application (0, 3, 12, and 24 h, Project Accession: PRJNA310171).

Ethephon is an ethylene generator which is widely used in *Hevea* to stimulate latex flow, and hence rubber yield. The mechanisms of ethylene signal transduction and yield stimulation are, as yet, poorly understood. Since the CBL-CIPK complex plays an important role in calcium signaling in relation to various plant responses (Luan, 2009), we examined the expression levels of *HbCBL* and *HbCIPK* genes in latex upon ethephon treatment. As shown in Figure 6, *HbCIPK14-16* and *27* and *HbCBL10* were obviously up-regulated following the ethephon treatment, while *HbCIPK7* and *HbCBL3* were down-regulated. Expressions of *HbCIPK11* and *30* displayed a transient high expression at 12 h after ethephon treatment but weakly expressed at 24 h. The expressions of *HbCBL1, 3* and *8* and *HbCIPK10, 14–16, 18* and *27* were further investigated by qPCR (Supplementary Figure 3); all showing patterns consistent with those of the transcriptome analysis. The above results implicated the *HbCIPK*-*HbCBL* complexes in ethylene-simulated latex production.

### Expression Analysis of HbCIPK and HbCBL Genes in Response to the Treatment of Latex Tapping

**FIGURE 7:**
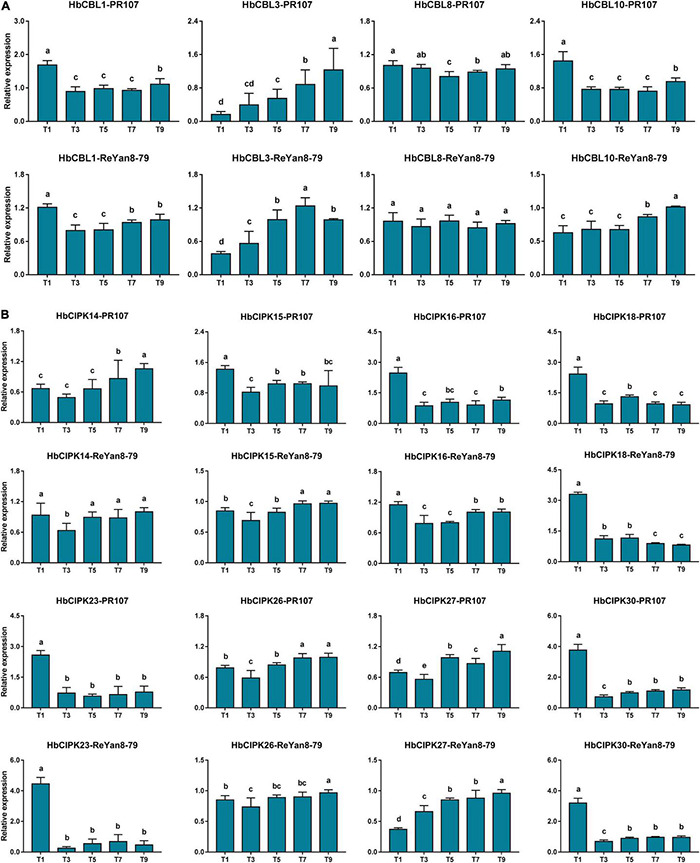
Expression analyses of *HbCIPK* and *HbCBL* genes in latex of trees newly brought into tapping based on qPCR. **(A)** Expression of *HbCBL1, 3, 8*, and *10* transcripts in latex from the first, third, fifth, seventh, and ninth tappings (T1, T3, T5, T7, and T9) of previously untapped *Hevea* trees of the clones PR107 and Reyan8-79. **(B)** Expression of *HbCIPK14, 15, 16, 18, 23, 26, 27*, and *30* transcripts in the first, third, fifth, seventh, and ninth tappings (T1, T3, T5, T7, and T9) of previously untapped *Hevea* trees of the clones PR107 and Reyan8-79. Values are means and standard deviations of three biological replicates. Different letters indicate significant differences with *P* < 0.05.

To assess the respective effect of latex tapping on the expression of *HbCIPK* and *HbCBL* genes, experiments were performed on previously untapped cultivated trees of the clones PR107 and ReYan8-79, and a number of *HbCIPK* and *HbCBL* genes with substantial expression in latex were examined. The first tapping of an untapped rubber tree normally produces very little latex. The latex yield increases progressively with successive tappings at regular intervals to reach a steady output after seven to ten tappings (Tang C. et al., 2010). As shown in Figures 7A,B, the expressions of *HbCIPK27* and *HbCBL3* were clearly up-regulated when untapped PR107 and ReYan8-79 trees were first brought into tapping. On the other hand, *HbCIPK16, 18, 23, 30 and HbCBL1* were significantly down-regulated from the second tapping onward. The above results suggested that the CIPK-CBL complexes might play a role in tapping-stimulated latex production. The expression patterns of *HbCIPK* and *HbCBL* genes in the two Hevea clones were consistent in most cases, indicating the ways of their response to the tapping treatment being similar under different *Hevea* genetic backgrounds.

### Interaction Analyses of HbCBL and HbCIPK Proteins

**FIGURE 8:**
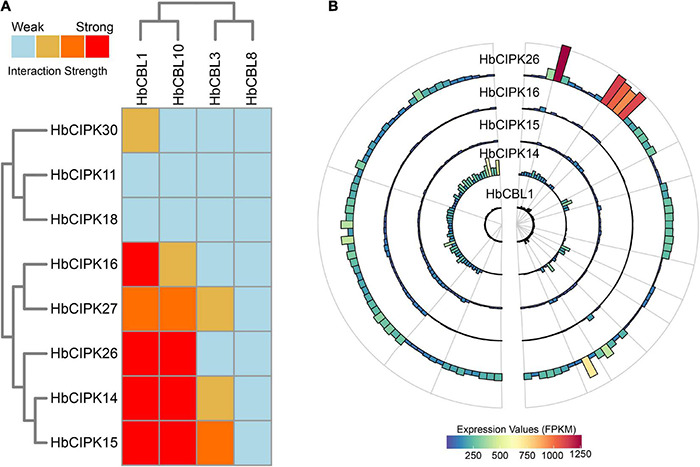
Interaction and expression patterns of CBL and CIPK families in *Hevea brasiliensis*. **(A)** Heat map summarizing yeast two-hybrid (Y2H) results for the HbCBL and HbCIPK combinations. The CIPKs and CBLs were fused, respectively, to activation domain (AD) and DNA-binding domain (BD) of a split transcription factor and screened for interactions between CIPK-AD/CBL-BD fusion proteins. Interaction strength was determined by serial growth dilutions on different types of selective media as detailed in Materials and Methods and summarized qualitatively by heat map. Red boxes indicate vigorous growth on -LTHA plates; orange boxes indicate weaker growth on -LTHA plates; yellow boxes indicate robust growth on -LTH plates but no growth on -LTHA plates; light blue boxes indicate weak or no obvious growth on -LTH plates. The Y2H images of each assay were shown in Supplementary Figure 4. **(B)** Bar plots showing expression profiles of *HbCBL1* and the corresponding strong Y2H-interacting *HbCIPKs*, *HbCIPK14*, *HbCIPK15*, *HbCIPK16*, and *HbCIPK26* in different tissues and treatments based on Solexa sequencing.

**FIGURE 9:**
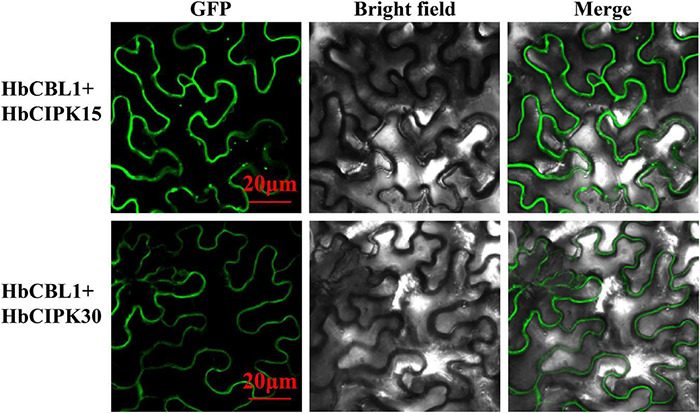
*In vivo* BiFC analysis of interaction between HbCBL1 and two HbCIPKs, HbCIPK15 and HbCIPK30 co-expressed in *N. benthamiana* leaf cells. The coding regions of HbCIPKs and HbCBL1 were fused to the N- and C-terminal halves of YFP, respectively. The fluorescence of YFP formed by the indicated plasmid combinations was observed 4 days after infiltration in *N. benthamiana* leaves by confocal laser microscopy. Bar = 20 μm.

Calcineurin B-like proteins and calcineurin B-like interacting protein kinases are functionally linked through physical interactions, and participate in plant development various environmental stresses (Luan, 2009). The interaction relationships between the HbCBL and HbCIPK proteins with substantial gene expression in latex or other *Hevea* tissues were detected by yeast two-hybrid (Y2H), i.e., spotting co-transformed yeast cells on various types of selective media. As shown in Figure 8A and Supplementary Figure 4, multiple combinations of HbCBL and HbCIPK fusion proteins showed obvious interactions although the strength of interaction varied. HbCBL1 and its closely related homolog, HbCBL10, displayed overlapping strong or moderate interactions with more than half of the HbCIPKs examined. In contrast, HbCBL3 and its close homolog HbCBL8 did not interact with most of the HbCIPKs investigated. Similar overlapping patterns of interaction were observed for the closed related HbCIPK homologs, e.g., HbCIPK11 and 18, and HbCIPK14 and 15. Expression profiles of the genes encoding HbCBL1 and its four strong interaction HbCIPK partners revealed by the Y2H assay (Figure 8A), HbCIPK14, HbCIPK15, HbCIPK16, and HbCIPK26, were examined in different tissues and treatments based on Solexa sequencing (Figure 8B and Supplementary Table 4). The expressions of *HbCIPK26* and *HbCBL1* displayed a strong correlation in different tissues and treatments, particularly in latex. To validate the results of the Y2H assay, the interactions of HbCBL1-HbCIPK15 (strong interaction) and HbCBL1-HbCIPK30 (weak interaction) were further investigated by the BiFC experiments conducted in *N. benthamiana* leaves. As shown in Figure 9, the results were consistent with those of the Y2H analysis, with the co-transformation of the HbCBL1-HbCIPK15 pair yielding a stronger green fluorescence than that of the HbCBL1-HbCIPK30 pair.

## Discussion

The CBL-CIPK network has been reported in Arabidopsis, poplar, cassava, and rice as contributing to plant development and stress response, but has never been reported in *Hevea*. In this study, *HbCBL* and *HbCIPK* genes were identified and the evolutionary relationship, gene structure, chromosomal location, and tissue-specific expression were analyzed. In addition, the expression profiles during *Hevea* leaf development, following ethylene induced latex flow and initiation of trees into latex tapping have been examined.

Conserved domain analysis showed domains of kinase, NAF, and EF hand harbored in the twelve *HbCBL* and thirty *HbCIPK* genes (Figure 2), indicating the *Hevea* CBL and CIPK families with basic characteristics similar to those in Arabidopsis, poplar and cassava (Yu et al., 2007; Mo et al., 2018). These genome-wide identification results suggested that the number of CIPK genes detected in *Hevea* was close to that of *Populus* and cassava, and larger than that of *Ricinus*. The phylogenetic and amplification patterns of family members showed a clustering of HbCIPK26, 28, and 29 in the phylogenetic tree. Combining the data of chromosomal location, it can be deduced that the gene amplification of *HbCIPK28* and *HbCIPK29* was due to tandem replication, while *HbCIPK26* and *HbCIPK28* were amplified through chromosome replication (Figures 1A, 4). The structural analysis suggested that most *HbCIPK* genes contain one or two exons, although a small number had multiple exons (Group A, Figure 2A). Chromosomal location analysis revealed the twelve *HbCBL* and thirty *HbCIPK* genes mapped onto fourteen of the eighteen *Hevea* chromosomes. However, the distribution of these genes in the chromosomes was uneven, indicative of species evolution and genetic variation (Figure 4).

**FIGURE 10:**
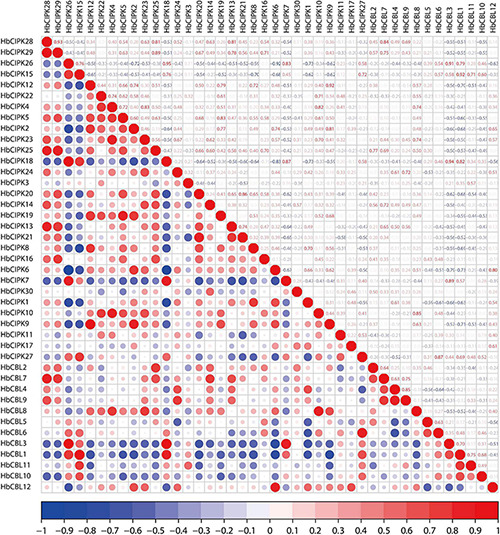
Correlation analyses of HbCIPK and HbCBL genes based on RNA-seq. Red indicates positive correlation and blue indicates negative correlation.

*HbCBL* and *HbCIPK* genes displayed different expression tendencies in different plant tissues (Figure 4). In latex, there were three *HbCIPK* members, *HbCIPK15, 18* and *26* that displayed relatively high expression levels, while only one *HbCBL* member, *HbCBL3*, was far more active in latex than the rest, which is consistent with the expression correlation analysis (Figure 10). It is speculated that *HbCIPK15, 18* and *26* interacts with *HbCBL3* for signal transmission in the latex functioning in the wounding response due to the tapping, latex flow stimulation by ethylene and rubber biosynthesis to regenerate rubber loss through tapping. More than half of the thirty *HbCIPK* family members were expressed during leaf development, suggesting an important role of the CBL-CIPK complex in the leaf growth and development of rubber tree.

Plants have developed unique strategies to adapt to adverse environments, and CBL-CIPK pathways participate in plant responses in this regard through environmental stress signals (Glazebrook, 1999; Luan, 2009). The mechanisms of latex flow upon tapping and ethylene yield stimulation in the rubber tree are not as yet fully understood (Tang C. et al., 2016). Certainly, these horticultural manipulations appear to invoke responses of some *HbCBL* and *HbCIPK* genes (Figures 6, 7). Most of the *HbCBL* and *HbCIPK* genes involved in these responses, whether up-regulated or down-regulated, might participate in some form of stress responses (Xiang et al., 2007; Hu et al., 2015; Mo et al., 2018). The expression trends in *HbCBL3* and *HbCIPK7, 18*, and *26* suggested that these genes play a role in sustaining the production of latex when trees are tapped routinely, but were negatively regulated by ethephon stimulation. On the other hand, *HbCBL1, 10* and *HbCIPK11, 14–16, 27*, and *30* respond positively to ethephon stimulation, associated possibly with subsequent increased latex output (Figure 6).

The set of *HbCBL* and *HbCIPK* genes responded differently when previously untapped trees were first brought into tapping (Figure 7). From the third tapping on, the activity of *HbCBL1* could be seen to have declined sharply. This was also the case for *HbCIPK16, 18, 23* and *30.* On the other hand, bringing the tree into tapping resulted in the expressions of *HbCBL3* and *HbCIPK26, 27* significantly up-regulated until the 9th tapping, indicating that the different genes had distinct functions when the tree starts latex production (Figure 7). The results also showed the mechanisms behind initiating latex flow in a previously untapped tree differed from those involved in increasing flow by ethylene stimulation in a tree already subjected to regular tapping.

In Arabidopsis, the SOS pathway is well defined as a salt tolerance signaling pathway that contains three key components, i.e., CPL4/SOS3, CIPK24/SOS2 and the plasma membrane Na^+^/H^+^ antiporter SOS1 (Quan et al., 2007). This pathway is conserved in other plants, e.g., rice, poplar, pear and an *Euphorbiacae* relative of *Hevea*, cassava, and the SOS components from distantly related plants could form inter-species protein complexes and confer salt tolerance to co-transformed yeast cells (Martinez-Atienza et al., 2007; Tang R. et al., 2010; Tang J. et al., 2016; Mo et al., 2018). To determine whether the HbCPL-HbCIPK complexes are involved in the SOS pathway mediated salt tolerance, different combinations of *HbCBLs* and *HbCIPKs* were co-transformed with *HbSOS1* or *AtSOS1* in a yeast mutant strain AXT3K. However, co-expression of any of the three-gene combinations did not confer better salt tolerance to transformed yeast cells than any of the two-gene combinations (HbCIPKs-HbSOS1 and HbCIPKs-AtSOS1) or single gene (HbSOS1 or AtSOS1) transformed cells (Supplementary Figure 5). In cassava, the MeCBL10-MeCIPK24-MeSOS1 complex forms a functional SOS pathway that confers salt tolerance to co-transformed yeast cells (Mo et al., 2018). Phylogenetic analysis revealed HbCBL8 and HbCIPK30 as the orthologs of MeCBL10 (named as MeCBL1 in this study) and MeCIPK24, respectively (Figure 1). Unexpected, HbCBL8 and HbCIPK30 revealed no physical interaction (Figure 8), indicating a possible discrepancy in forming an active SOS pathway in *Hevea*.

In summary, we conducted a genome-wide survey of the *HbCBL* and *HbCIPK* gene families in *Hevea*. A total of twelve *CBL* and thirty *CIPK* genes were identified in the *Hevea* genome database and divided into four and five groups, respectively, by the phylogenetic comparison of homologous genes from *Hevea* and five other plant species. The genes were assigned to the *Hevea* chromosomes and their putative ways of evolution were suggested. Expressional analysis among various *Hevea* tissues and phases of leaf development revealed their diversified spatiotemporal expression patterns. Finally, our transcript analysis of *HbCBL* and *HbCIPK* genes following ethylene yield stimulation and the induction of latex tapping, together with the results of multiple HbCBL and HbCIPK interactions, suggested the involvement of CBL-CIPK complexes in responding to many forms of stresses that impact on rubber yield. Our results of genome-wide identification, expression analysis and protein interaction detection provide a foundation for further functional research on the CBL-CIPK pathways in *Hevea*.

## Materials and Methods

### Identification of Hevea Calcineurin B-Like Interacting Protein Kinases and Calcineurin B-Like Proteins Genes

Sequences of *Arabidopsis thaliana*, *Populus trichocarpa*, and *Oryza sativa* CBL and CIPK genes were downloaded from GenBank^1^. The genome and protein sequences of *Arabidopsis thaliana*, *Oryza sativa*, *Populus trichocarpa*, *Manihot esculenta*, and *Ricinus communis* were downloaded from Phytozome^2^. The *Hevea* Genome and transcriptome were obtained from GenBank^3^. Local BLAST and Hidden Markov Model searches were conducted to identify *Hevea* CBLs and CIPKs using Arabidopsis, rice and poplar CBL and CIPK protein sequences as queries to search against the proteome of each species for the candidate *CIPKs* and *CBL*s from *H. brasiliensis*, *Arabidopsis thaliana, Oryza sativa, Populus trichocarpa, Manihot esculenta*, and *Ricinus communis.* All putative candidates were manually verified with the InterProScan server^4^ to confirm the presence of relevant protein domains. The website ProtParam^5^ was used to predict protein isoelectric point and molecular weight.

### Phylogenetic, Gene Structure and Chromosomal Location Analyses of Calcineurin B-Like Interacting Protein Kinases and Calcineurin B-Like Proteins Genes

Multiple alignments of the amino acid sequences of *CIPK* and *CBL* from *Hevea brasiliensis* and five other species were set up and phylogenetic trees were constructed with MEGA6.0 by employing the Neighbor-Joining (NJ) method with a bootstrap test for 1,000 replicates. Exon/intron structures of *HbCBL* and *HbCIPK* genes were analyzed by comparing the cDNA and their genomic DNA sequences, and chromosomal locations analyzed through mapping the genes to chromosomes, both using the TBtools software (Chen et al., 2020). The collinearity analysis was completed using MCscanX software (Wang et al., 2012).

### Expression Analysis Based on Solexa Sequencing

The Solexa sequencing data for various tissues/organs, developmental stages and ethephon stimulation available at the NCBI Sequence Read Archive (SRA) database were used for the expression analysis of *Hevea brasiliensis* as described previously (Project Accession: PRJNA310171, Supplementary Table 3) (Xiao et al., 2017). The *Hevea* tissues included (a) latex, bark, leaf, root, seed, female flower and male flower, (b) leaves of four developmental stages (bronze, color-change, pale-green and mature), and (c) latex samples collected at 0, 3, 12, and 24 h after ethephon stimulation. Raw RNA-seq reads were processed to trim terminal low-quality bases and adapter sequences via an in-house custom pipeline. The clean reads were then mapped to the *Hevea brasiliensis* genome using Bowtie2, and RSEM software was used for quantifying transcript abundance with default parameters (Li and Dewey, 2011). The normalized value expression profiles of *HbCBL1* and its interaction partner genes were visualized in bar-plots by R^6^.

### RNA Isolation and Quantitative Real-Time PCR

To verify the results obtained by Solexa sequencing and to examine genes expression, qPCR was performed on a number of the *Hevea brasiliensis CIPK* and *CBL* genes as described previously (Li et al., 2011). The primer pairs used for the *CIPK* and *CBL* genes were listed in Supplementary Table 1. For internal control, the genes RH2b and YLS8 were used as described previously (Li et al., 2011).

### Correlation Analysis Based on Gene Expression

To analyze the relationship between *CBL* and *CIPK* family members, the expression correlations among family members were analyzed. The input data of correlation analysis was the FPKM values of RNA-seq, included the *Hevea* (a) tissues latex, bark, leaf, root, seed, female flower and male flower, (b) leaves of four developmental stages (bronze, color-change, pale-green and mature), and (c) latex samples collected at 0, 3, 12, and 24 h after ethephon stimulation. R package Corrplot^7^ was used for analysis and drawing.

### Yeast Two-Hybrid Assay and Yeast Complementation Test

The full length cDNA of eight *HbCIPK* and four *HbCBL* genes with substantial expressions in latex were amplified by PCR with their respective primers (Supplementary Table 1) and inserted into the Y2H vectors of pGADT7 and pGBKT7, respectively. The pGBKT7-HbCBLs and pGADT7- HbCIPKs vectors were co-transformed into the Y2HGold yeast (*Saccharomyces cerevisiae*) strain and examined for their interactions using the MatchMaker yeast two-hybrid system (Clontech, United States). The detailed protocols were as described (Kleist et al., 2014). To test the functionality of the latex expressed HbCBL-HbCIPK complexes in salt tolerance SOS pathway, complementation test was conducted using the yeast mutant strain AXT3K (4ena1:HIS3:4ena4,4nha1:LEU2, and 4nhx1:KanMX4) that lacks the main plasma membrane Na^+^ transporters (Quintero et al., 2011; Zhou et al., 2015). The full length coding regions of *HbCBL1/8* and *HbCIPK14/15/27/30* were amplified by PCR (Supplementary Table 1) and cloned into the yeast expression vector p414 (Mo et al., 2018), whereas the coding regions of HbSOS1 and AtSOS1 were cloned into the yeast expression vector p416 (Yin et al., 2020). The three plasmids (p414-*HbCBLs*, p414-*HbCIPKs*, and p416-*HbSOS1* or *AtSOS1*) were co-transformed into the yeast strain, and the salt tolerance tests were performed according to the detailed process as described previously (Yin et al., 2020).

### Bimolecular Fluorescence Complementation Assay

Open reading frames (ORFs) of HbCIPK15, HbCIPK30 and HbCBL1 were amplified by RT-PCR and cloned into the pEG100-YFP vectors to construct fusion proteins at the N- and C-termini. Primers are listed in Supplementary Table 1. Each cDNA was under the control of the 35S promoter. For transient expression in *Nicotiana benthamiana* leaves, plasmids were introduced into *Agrobacterium tumefaciens* strain GV3101 competent cells for infiltration of 5-week-old *N. benthamiana* leaves. For microscopic observation, the reconstructed green fluorescence protein (GFP) signals of the lower epidermal cells of leaves cut 4 day after infiltration were examined using a Nikon A1RHD25 confocal microscope (Nikon, Japan).

## Data Availability Statement

The datasets presented in this study can be found in online repositories. The names of the repository/repositories and accession number(s) can be found below: https://www.ncbi.nlm.nih.gov/genbank/, PRJNA310171.

## Author Contributions

CT, XX, and YF conceived and designed the experiments. XX, CM, JS, XLi, XLo, YQ, and YF performed the experiments. XX, JS, and YF analyzed the data. XX and CT wrote the manuscript. All authors read and approved the final manuscript.

## Conflict of Interest

The authors declare that the research was conducted in the absence of any commercial or financial relationships that could be construed as a potential conflict of interest.

## Publisher’s Note

All claims expressed in this article are solely those of the authors and do not necessarily represent those of their affiliated organizations, or those of the publisher, the editors and the reviewers. Any product that may be evaluated in this article, or claim that may be made by its manufacturer, is not guaranteed or endorsed by the publisher.

## Footnotes

1
http://www.ncbi.nlm.nih.gov/genbank

2
https://phytozome-next.jgi.doe.gov/

3
http://www.ncbi.nlm.nih.gov/nuccore/448814761

4
http://www.ebi.ac.uk/Tools/pfa/iprscan/

5
https://web.expasy.org/protparam/

6
https://www.r-project.org/

7
https://cran.r-project.org/web/packages/corrplot/index.html
