# Supplementary material for: The Calcium Sensor Calcineurin B-Like Proteins -Calcineurin B-Like Interacting Protein Kinases Is Involved in Leaf Development and Stress Responses Related to Latex Flow in Hevea brasiliensis
Source: Front Plant Sci. 2022 Feb 25;13:743506. doi: 10.3389/fpls.2022.743506 (PMC8914471; doi:10.3389/fpls.2022.743506)
Supplement: Supplementary file 5 [file Data_Sheet_1.doc]

**Figure S1**


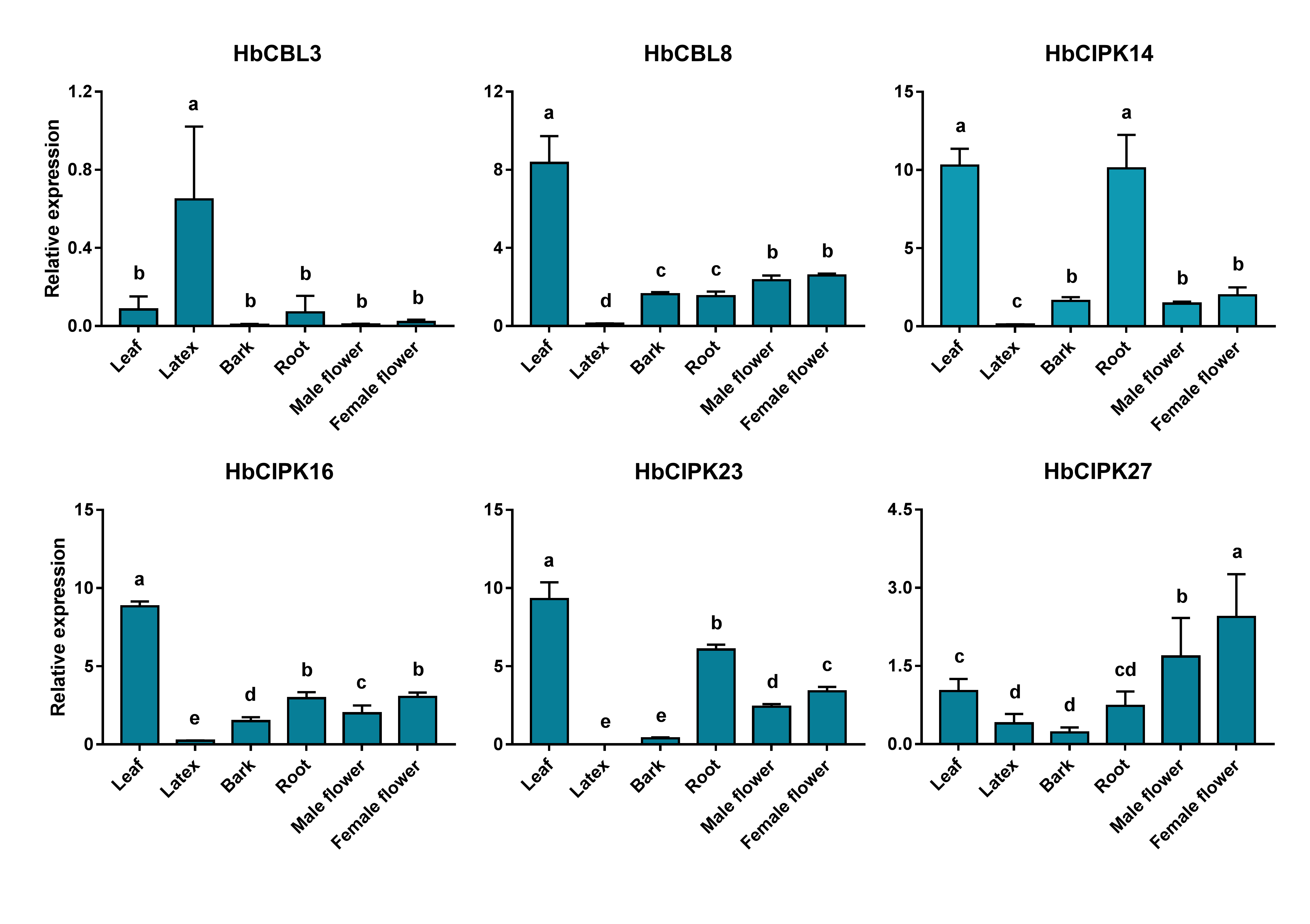


**Figure S1.** Expression analyses of *HbCIPK* and *HbCBL* genes in different tissues based on qPCR. Relative expressionvalues of *HbCIPK14, 16, 23, 27* and *HbCBL3, 8* in seven tissues (leaf, bark, latex, root, seed, female flower, and male flower) were given as means ± standard deviations of three biological replicates. Different letters indicate significant difference with P < 0.05.

**Figure S2**


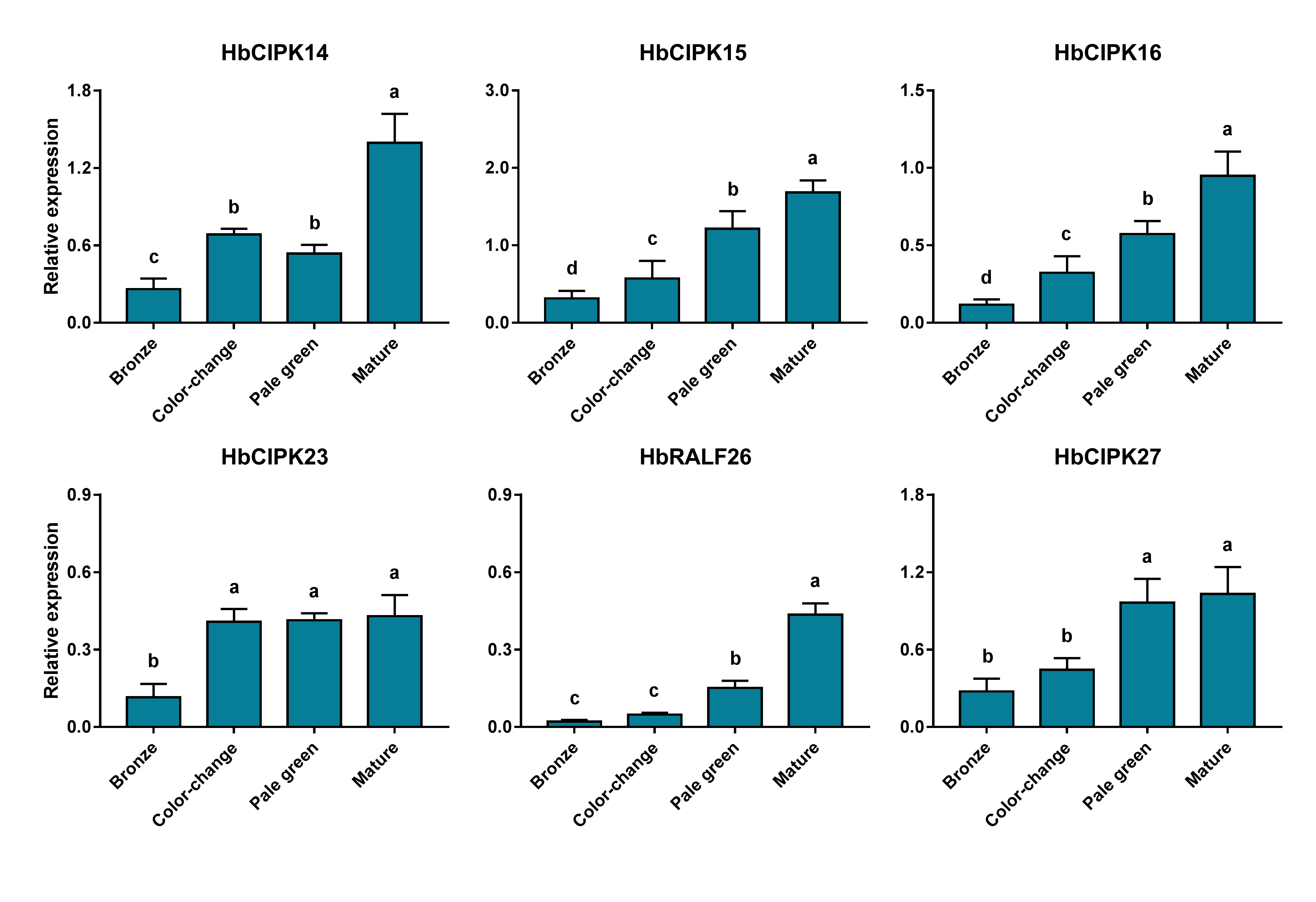


**Figure S2.** Expression analyses of six *HbCIPK* genes at different leaf developmental stages based on qPCR. Relative expression values of *HbCIPK14, 15, 16, 23, 26,* and *27* at four developmental stages of leaf (bronze, color change, pale-green and mature) were given as means ± standard deviations of three biological replicates. Different letters indicate significant difference with *P* < 0.05.

**Figure S3**


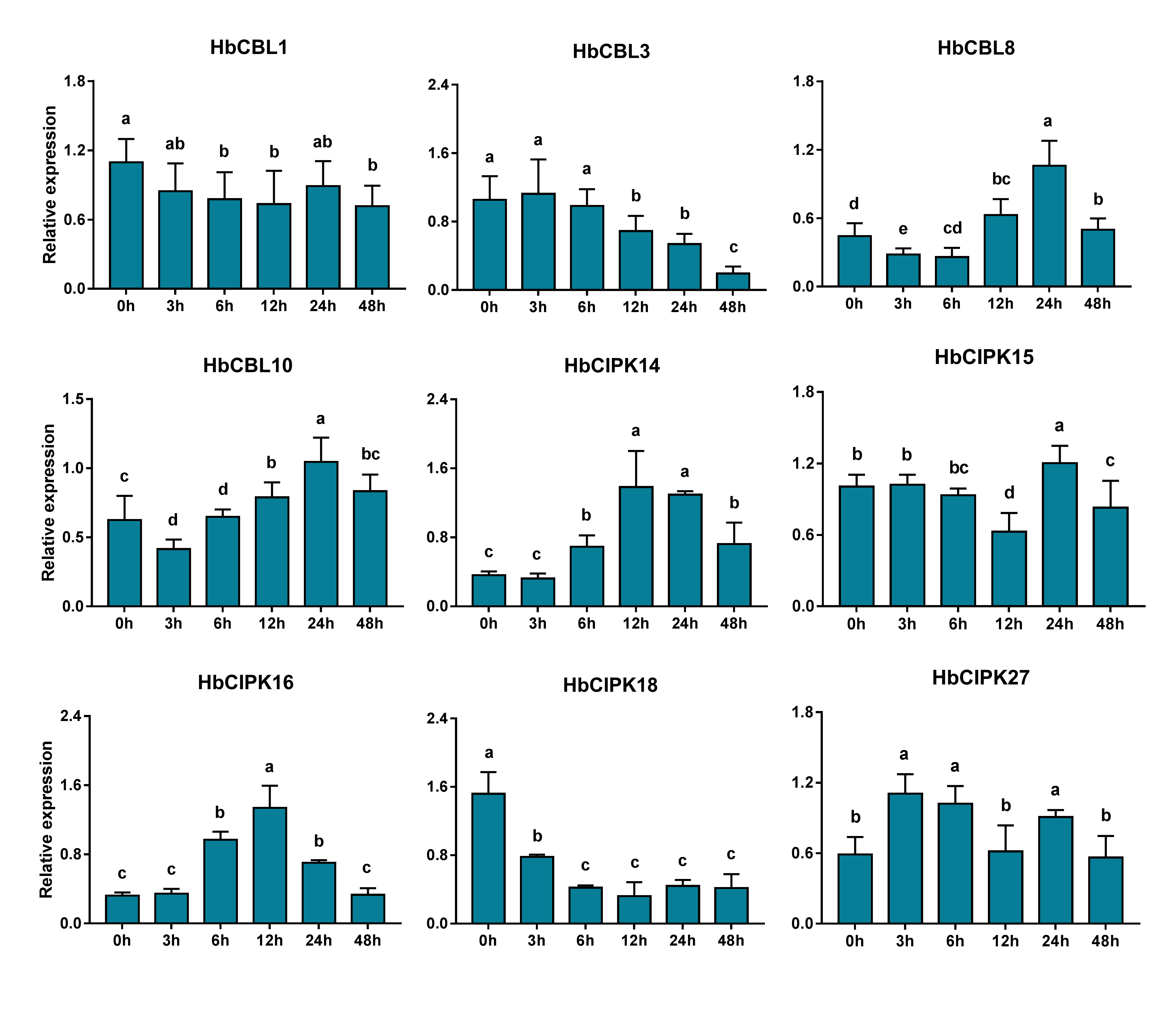


**Figure S3.** Expression analyses of *HbCIPK* and *HbCBL* genes in latex following ethephon treatment based on qPCR. Relative expression values of *HbCBL1, 3, 8*, *10* and *HbCIPK14, 15, 16, 18 and 27* transcripts in latex following ethephon treatment (0 h, 3 h, 6 h, 12 h, 24 h, and 48 h) were given as means ± standard deviations of three biological replicates. Different letters indicate significant difference with *P* < 0.05.

**Figure S4**


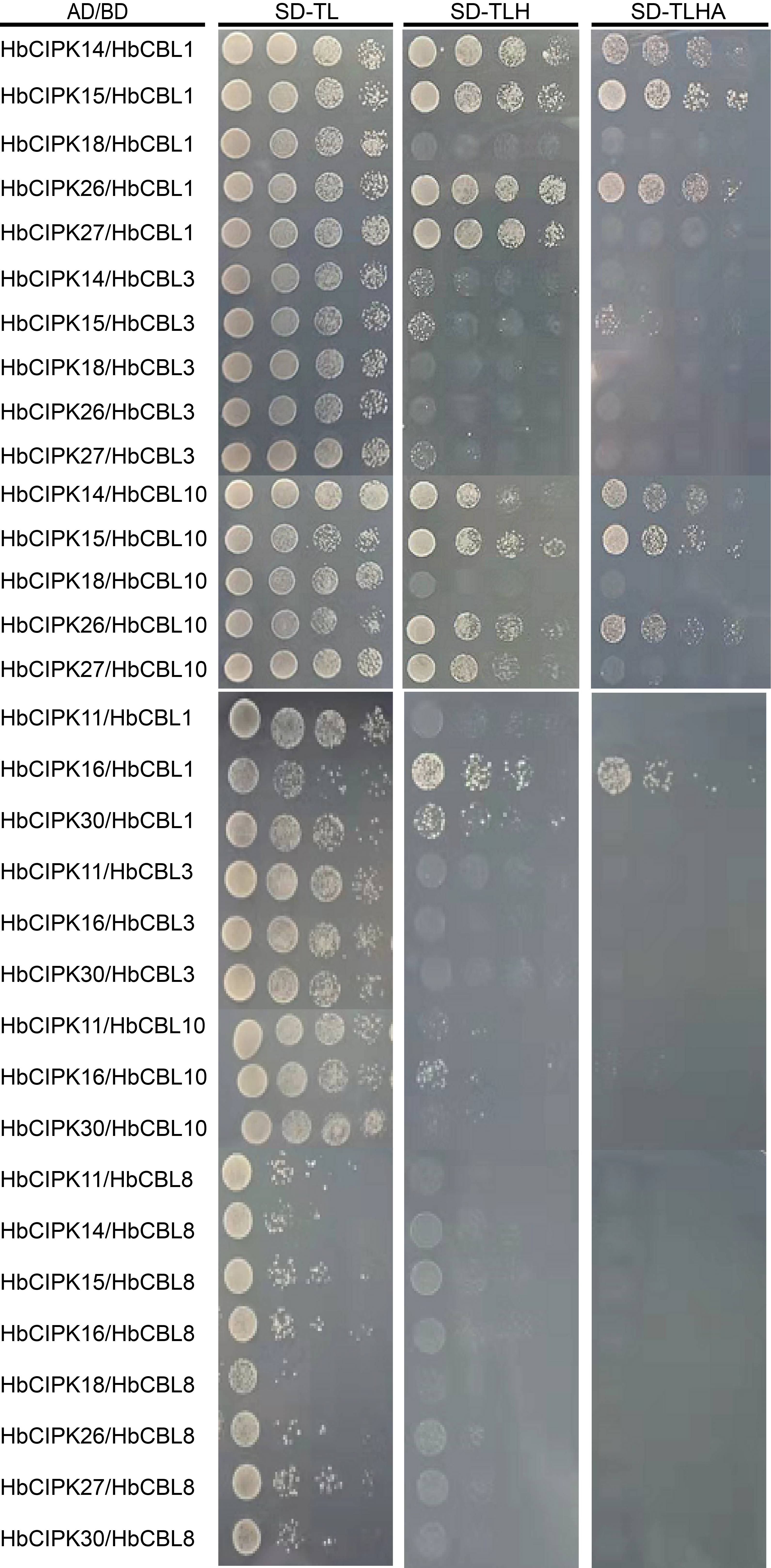


**Figure S4.** Identifying interactions between HbCIPK and HbCBL proteins. Interactions between HbCIPK (HbCIPK11/14/15/16/18/26/27/30) and HbCBL (HbCBL1/3/8/10) proteins were assayed using a yeast two-hybrid method. Yeast cells harboring different AD- and BD-fusion protein combinations were plated on SD-TL (lacking Trp and Leu), SD-TLH (lacking Trp, Leu and His), and SD-TLHA (lacking Trp, Leu, His and Ade) media, as described in the section “Materials and Methods”, and cultured at 28℃ for 3-5 days.

**Figure S5**


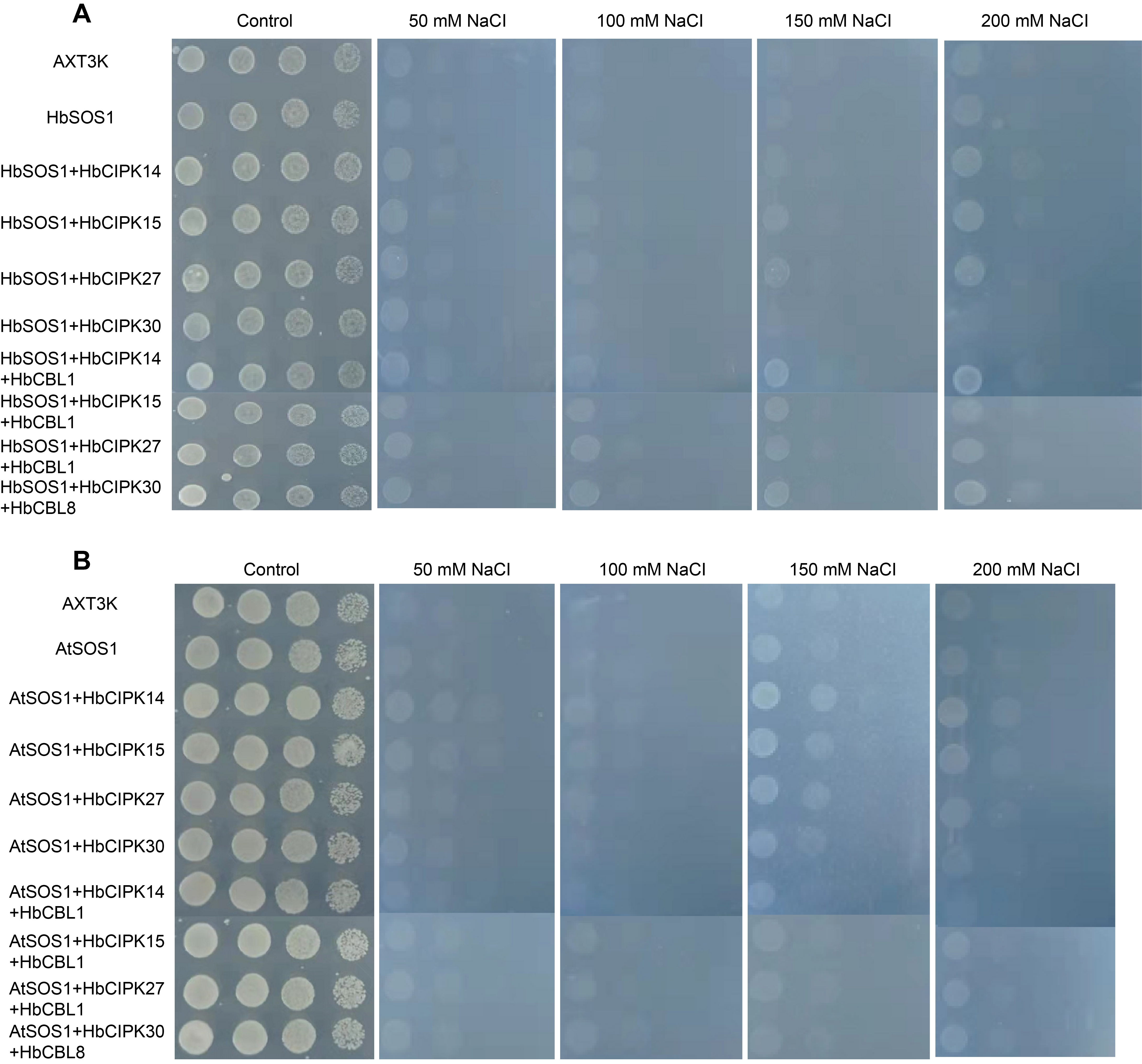


**Figure S5.** Yeast complementation test for salinity stress tolerance. (A), transformed and untransformed yeast cells were cultured to saturation, and serially diluted 10-fold and spotted on AP plates without or with NaCl as detailed in Materials and Methods. After 5 days, the growth of yeast cells on plates was recorded. AXT3K, untransformed yeast mutant strain lacking salt tolerance capacity; HbSOS1, AXT3K strain transformed with the *Hevea* Na^+^/H^+^ antiporter gene *HbSOS1*; HbSOS1+HbCIPK, AXT3K strain transformed with *HbSOS1* and *HbCIPK* (*HbCIPK14/15/27/30*) genes; HbSOS1+HbCIPK+HbCBL, AXT3K strain transformed with *HbSOS1*, *HbCIPK* (*HbCIPK14/15/27/30*) and *HbCBL* (*HbCBL1/HbCBL8*) genes. (B), Same as (A) except for the replacement of *HbSOS1* with its *Arabidopsis* ortholog *AtSOS1*.
